# Epidemiology of COVID-19 and Predictors of Outcome in Nigeria: A Single-Center Study

**DOI:** 10.4269/ajtmh.20-0759

**Published:** 2020-10-26

**Authors:** Olayinka Rasheed Ibrahim, Bello Muhammed Suleiman, Suleiman Bello Abdullahi, Taofeek Oloyede, Abdallah Sanda, Maruf Sanusi Gbadamosi, Bashir Olajide Yusuf, Rabilu Yandoma Iliyasu, Lawal Magaji Ibrahim, Adamu Danladi Dawud, Sulaiman Saidu Bashir, Nwawueze Efam Okonta, Wasinda Francis Umar, Abiodun Gbenga Tekobo, Muhammadu Sani Abubakar, Bashir Taiye Aminu, Shuaibu Onoruoyiza Ibrahim, Rasaq Olaosebikan, Olugbenga Ayodeji Mokuolu

**Affiliations:** 1 Department of Pediatrics, Federal Medical Center, Katsina, Nigeria;; 2 Department of Family Medicine, Federal Medical Center, Katsina, Nigeria;; 3 Department of Internal Medicine, Federal Medical Center, Katsina, Nigeria;; 4 Department of Medical Microbiology, Federal Medical Center, Katsina, Nigeria;; 5 Department of Disease Control and Immunization, National Primary Health Care Development Agency, Abuja, Nigeria;; 6 Department of Community Medicine, Ahmadu Bello University Teaching Hospital, Amadu Bello University, Zaria, Nigeria;; 7 Department of Anesthesia, Federal Medical Center, Katsina, Nigeria;; 8 Department of Pharmacology and Experimental Pharmacology, Thomas Jefferson University, Philadelphia, Pennsylvania;; 9 Department of Pediatrics, University of Ilorin Teaching Hospital, University of Ilorin, Ilorin, Nigeria

## Abstract

There is a paucity of information regarding the epidemiology and outcome of COVID-19 from low/middle-income countries, including from Nigeria. This single-center study described the clinical features, laboratory findings, and predictors of in-hospital mortality of COVID-19 patients. Patients admitted between April 10, 2020 and June 10, 2020 were included. Forty-five patients with a mean age of 43 (16) years, predominantly male (87%), presented with fever (38%), cough (29%), or dyspnea (24%). In-hospital mortality was 16%. The independent predictors of mortality were hypoxemia (adjusted odds ratio [aOR]: 2.5; 95% CI: 1.3–5.1) and creatinine > 1.5 mg/dL (aOR: 4.3; 95% CI: 1.9–9.8).

## INTRODUCTION

The world is struggling with the COVID-19 pandemic, which broke out in Wuhan, Hubei Province, of China in December 2019.^1^ The novel SARS-CoV-2 that causes COVID-19 has overwhelmed the high-, middle-, and low-income countries.^2^ COVID-19 is the greatest challenge facing humans with more than 34.8 million cases and more than a million deaths as stated in the WHO situation report on October 5, 2020.^3^ The situation report in Africa (September 29, 2020) also showed 1,465,023 COVID-19 cases and 35,750 (case fatality rate: 2.4%) deaths in 55 African countries inclusive of Nigeria.^4^ Similarly, the number of cases in Nigeria has risen to 59,465 after the index case on February 27, 2020 with 1,113 deaths (October 5, 2020).^5^

The amount of the literature available on COVID-19 has also risen in the past few months, spanning various aspects of the disease, including the epidemiological profile, manifestations, and outcomes.^6^ Studies showed the spectrum of illness from asymptomatic disease to respiratory failure to multiple-organ dysfunction in critically ill cases.^7^ In China, common clinical manifestations found in patients with COVID-19 included fever, cough, breathlessness, vomiting, and diarrhea.^8^ Reports from the United States also showed a similar spectrum of presentation in the non-severe cases.^9^ Patients with COVID-19 have abnormalities in laboratory parameters that include leukopenia and lymphopenia. Some series reports derangement in liver function tests, elevated blood urea, and serum creatinine.^10^ Besides, a study also found elevated levels of dimers and other biomarkers of acute inflammation.^11^ The outcomes of asymptomatic to moderate COVID-19 are good with most patients recovering with no sequel.^12^ By contrast, mortality from severe and critical cases may be as high as 45%, especially in the presence of end-organ damage and multiple-organ dysfunction syndrome (MODS).^13^ Besides, older age categories above 65 years and underlying medical conditions, including hypertension, diabetes, cardiovascular disease, obesity, and chronic obstructive pulmonary disease, were associated with poor outcomes.^14^

Despite the increasing available literature on COVID-19, very few publications have emerged from Africa inclusive of Nigeria.^15,16^ Besides, curtailing the COVID-19 pandemic in middle- and low-income countries requires understanding the clinical presentations, laboratory characteristics, and factors associated with poor outcomes in patients with COVID-19. We hypothesized that predictors of outcomes of COVID-19 in Nigeria may differ from those in other countries. To test this hypothesis, we described the epidemiology and outcomes of COVID-19 in a single-center cohort of patients. We determined which factors have an independent association with mortality.

## MATERIALS AND METHODS

This study was a retrospective review of cases of confirmed COVID-19 admitted to the hospital isolation and treatment center, Federal Medical Center, Katsina, Nigeria, from April 10, 2020 to June 10, 2020. All the cases of SARS-CoV-2 infection were confirmed using a real-time PCR (RT-PCR) test of nasopharyngeal and oropharyngeal samples at the National Reference Laboratory. We obtained ethical approval from the state/hospital which granted a waiver for patients’ consent (MOH/ADM/SUB/1152/1/373). The data were also anonymous with the absolute confidentiality of the patients.

The hospital is a tertiary health facility in the northwestern part of Nigeria. It has a dedicated treatment center with a full complement of staff and managed those with moderate, severe, and critical cases of COVID-19.

The retrospective study included all adults aged 18 years and older managed during the study period. We excluded children and adolescents younger than 18 years. We classified the patients, at admission, into asymptomatic, mild, moderate, severe, and critical cases using the China guideline for COVID-19 version 4. In brief, an asymptomatic patient was anyone without symptoms. Mild cases included those with complaints such as fever and cough with no evidence of systemic illness. Moderate cases included patients with high-grade fever, breathlessness, minimal supports, and oxygen saturation greater than 92% in room air. Severe cases were those patients with hypoxemia (oxygen saturation < 92%), who required supplemental oxygen, and in need of close monitoring. Critical cases included those patients who required intensive care admissions including ventilatory support, end-organ damage, or multiple-organ dysfunction.

The patients had routine investigations which included full blood count, liver function tests, electrolytes, urea and creatinine, random blood sugar, blood culture where indicated, and chest radiograph as part of the management protocol. All the patients received antiviral (lopinavir/ritonavir), vitamin C, zinc, and azithromycin. Besides, some patients received other medications based on their comorbid medical conditions. For instance, those with underlying hypertension and diabetes mellitus received antihypertensive and antidiabetic medications, respectively. Two patients received anti-tuberculosis (TB) because we confirmed them to have TB during their management. We managed the patients with the WHO standard protocol for case management; this comprised the use of high-flow oxygen with a re-breathable mask for those with hypoxemia and other supportive cares. Besides, two patients with respiratory failure received mechanical ventilation, and a patient with acute kidney injury had hemodialysis. All three patients were managed as critical COVID-19 and received intensive care unit (ICU) care. We followed national country guidelines in the discharge protocol of the patients. In brief, the criteria for discharge during the early part of the case management were based on two consecutive negative RT-PCR tests at least 48 hours apart. The discharge criteria were subsequently revised to a single negative result and at present based on the absence of fever for 3 days after at least 10 days of the initial positive results. In addition, the absence of symptoms and overall clinical satisfactory recovery, including normal saturation in room air, were part of the current discharge criteria.

### Definition of outcome.

The primary outcome of interest was hospitalization outcomes. The secondary outcomes included the description of the epidemiologic characteristics, clinical presentations, and laboratory findings of the COVID-19 cases.

### Data collection.

Aminu Bashir, Nwawueze Efam Okonta, and Muhammadu Sani Abubakar extracted the data from the electronic health record into an Excel spreadsheet. Olayinka Rasheed Ibrahim checked the data to ensure the accuracy of the information which included the demographics, clinical features, laboratory findings including the chest radiographic findings, and outcomes whether discharged or death.

The data were analyzed using Microsoft Excel and Statistical Package for Social Sciences (SPSS)™ for Windows version 20.0 (IBM Corp., Armonk, NY). The mean and SD were used to summarize normally distributed continuous variables, whereas the median with interquartile range (IQR) was used to summarize the skewed data. The discrete variables were summarized using frequency tables and percentages. We compared those who survived (at discharge) with those who did not (death) using chi-square and Fisher’s exact tests as appropriate. The continuous variables were compared using the *t*-test and Mann–Whitney *U* test for normally distributed and skewed data, respectively. A univariate analysis was carried out using odds ratio with 95% CI, whereas a multivariate logistic regression was carried out on some variables which included age, breathlessness, chest findings, pulse rate, hypoxemia, diabetes, neutrophils, and serum creatinine. We selected these variables based on the literature and *P*-values less than 0.05 on the univariate analysis. The multivariate analysis was reported as an adjusted odds ratio (aOR) with 95% CI. The level of statistically significant was a *P*-value less than 0.05.

## RESULTS

A total of 45 patients with the diagnosis of COVID-19 were managed during the study period at the Federal Medical Center, Katsina, northwestern Nigeria. The hospital is a tertiary health facility fairly well-equipped compared with most COVID-19 treatment centers in Nigeria. Despite being limited by bed spaces (15) for COVID-19, it has two functioning ventilators with the capacity to carry out mechanical ventilation and a dedicated hemodialysis machine for COVID-19.

**Figure 1.**
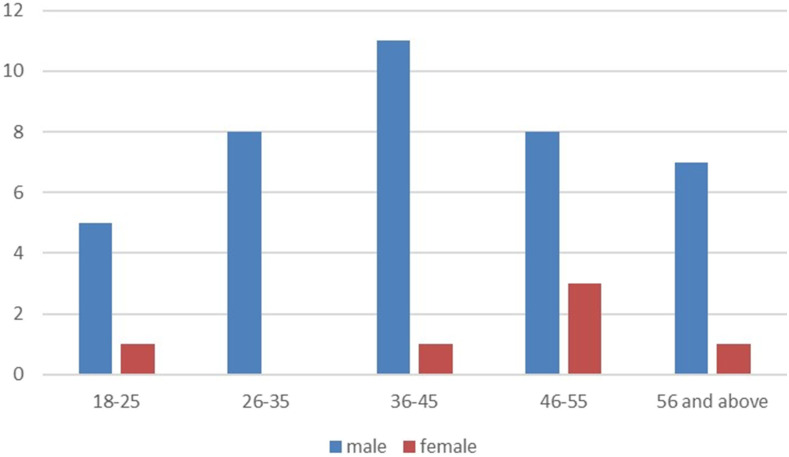
Age and gender distribution of the patients.

Patients were young (Figure 1) and predominantly male (Table 1). The predominant symptoms were fever (17, 37.8%) and cough (13, 28.9%) as shown in Table 2. Also, nine patients (20.0%) had underlying hypertension, and seven (15.6%) were obese. The median (IQR) duration of symptoms before presentation was 4 (2–8.5) days. Based on the index of severity (determined at the final diagnosis), most of the patients (21, 46.7%) were asymptomatic. Of 35 patients with complete data for full blood count, 14.3% had leukocytosis (Table 3).

**Table 1:** Demographic characteristics of the patients

Variable	Total, *n* (%)	Survivor, *n* (%)	Non-survivor, *n* (%)	*P*-value
Age (years)				
< 30	10 (22.2%)	10 (26.3%)	0	**0.022**
30–49	22 (48.9%)	20 (52.6%)	2 (28.6%)	
50–69	8 (17.8%)	4 (10.5%)	4 (57.1%)	
> 75	5 (11.1%)	4 (10.5%)	1 (14.3%)	
Mean (SD) years	43 (16.0)	40.8 (15.9)	55.0 (10.8)	**0.029**
Gender				
Male	39 (86.7%)	32 (84.2%)	7 (100%)	0.569
Female	6 (13.3%)	6 (15.8%)	(0)	
Total	45	38 (84.4)	7 (15.6)	

Bold indicates *P* values that are statistically significant.

**Table 2:** Clinical features of the patients (at admission)

Variable	Total, *n* (%)	Survivor, *n* (%)	Non-survivor, *n* (%)	*P*-value
Symptoms				
Fever	17 (37.8)	10 (26.3)	7 (100.0)	**< 0.001**
Cough	13 (28.9)	7 (18.4)	6 (85.7)	**0.001**
Breathlessness	11 (24.4)	6 (15.8)	5 (71.4)	**0.006**
Reduced appetite	8 (17.8)	4 (10.5)	4 (57.1)	**0.013**
Anosmia	6 (13.3)	4 (10.5)	2 (28.6)	0.230
Vomiting	4 (8.9)	2 (5.3)	2 (28.6)	0.108
Diarrhea	6 (13.3)	3 (7.9)	3 (42.9)	**0.039**
Body weakness	6 (13.3)	3 (7.9)	3 (42.9)	**0.039**
Signs				
Chest findings				
Normal	34 (75.6)	34 (89.5)	0	**< 0.001**
Abnormal	11 (24.4)	4 (10.5)	7 (100.0)	
Respiratory rate				
20 and below	21 (46.7)	20 (52.6)	1 (14.3)	0.101
> 20	24 (53.3)	18 (47.4)	6 (85.7)	
Pulse rate				
100 and below	35 (77.8)	33 (86.8)	2 (28.6)	**0.003**
> 100	10 (22.2)	5 (13.2)	5 (71.4)	
Systolic BP*				
140 and below	32 (71.1)	29 (76.3)	3 (42.9)	0.168
> 140	13 (28.9)	9 (923.7)	4 (57.1)	
Diastolic BP*				
90 and below	40 (88.9)	35 (92.1)	5 (71.4)	0.166
> 90	5 (11.1)	3 (7.9)	2 (28.6)	
Oxygen saturation†				
< 92%	13 (28.9)	8 (15.8)	5 (100.0)	**< 0.001**
92% and more	32 (71.1)	30 (84.2)	2	
Underlying medical condition				
Hypertension	9 (20.0.)	7 (18.4)	2 (28.6)	0.614
Diabetes mellitus	6 (13.3)	3 (7.9)	3 (42.9)	**0.039**
Obesity‡	7 (15.6)	2 (5.3)	5 (71.4)	**< 0.001**
Tuberculosis	2 (4.4)	1 (2.6)	1 (14.3)	0.290
Index of severity§				
Asymptomatic	21 (46.7)	21 (55.3)	0	**< 0.001**
Mild	9 (20.0)	9 (23.7)	0	
Moderate	4 (8.9)	4 (10.5)	0	
Severe‖	11 (24.4)	4 (10.5)	7 (100)	
Duration of hospitalization (days) median (interquartile range)	10 (6.0–14.5)	11 (9.0–15.0)	2.0 (1.3–5.0)	**< 0.001**
Total	45	38 (84.4)	7 (15.6)	

BP = blood pressure.

* mmHg.

† # Oxygen saturation at presentation.

‡ # Defined as body mass index ≥ 30 Kg/m^2^.

§ ≠ Final diagnosis at discharge.

‖ Inclusive of three critical cases.

**Table 3:** Laboratory findings in the patients at presentation

Variable	Total, *n* (%)	Survivor, *n* (%)	Non-survivor, *n* (%)	*P*-value
White blood count (× 10^9^/L)	35*	31	4	
< 3.5	1 (2.9)	1 (3.2)	0	**0.012**
3.5–10	29 (82.9)	28 (90.3)	1 (25.0)	
> 10	5 (14.3)	2 (6.5)	3 (75.0)	
Lymphocyte (%)	35*	31	4	
< 20	18 (51.4)	14 (45.2)	4 (100.0)	0.104
20–40	0	0	0	
> 40	17 (48.6)	17 (44.7)	0	
Neutrophils (%)	35*	31	4	
< 40	13 (37.1)	13 (41.9)	0	**0.012**
40–60	13 (37.1)	13 (41.9)	0	
> 60	9 (25.7)	5 (16.1)	4 (100.0)	
Hemoglobin (g/dL)	35*	31	4	
< 30	2 (5.7)	1 (3.2)	1 (25.0)	–
≥ 30	33 (73.3)	30 (96.8)	3 (75.0)	
Platelets (× 10^9^/L)	35*	31	4	
< 100	5 (14.3)	4 (12.9)	1 (25.0)	–
100–300	30 (85.7)	27 (87.1)	3 (75.0)	
Total protein (g/L)	30†	29	1	
< 60	1 (3.3)	1 (3.4)	0	1.000
60–80	29 (96.7)	28 (96.6)	1 (100.0)	
Albumin (g/L)	30†	29	1	
< 30	2 (6.7)	2 (6.9)	0	1.000
30–50	28 (93.3)	27 (93.1)	1 (100.0)	
Aspartate aminotransferase (IU/L)	30†	29	1	
Normal (< 45)	30 (100.0)	29 (100)	1 (100.0)	–
Elevated	0	0	0	
Alanine aminotransferase (IU/L)	30†	29	1	
Normal (< 45)	29 (96.7)	28 (96.6)	1 (100.0)	1.000
Elevated	1 (3.3)	1 (3.4)	0	
Serum creatinine (mg/dL)	35*	32	3	
≤ 1.5	33 (94.3)	32 (100)	1 (33.3)	**0.005**
> 1.5	2 (5.7)	0	2 (66.7)	
Chest radiography	11‡	7	4	
Ground glass	5 (45.5)	3 (42.9)	2 (50.0)	0.448
Patch opacities	3 (27.3)	1 (14.3)	2 (50.0)	
Normal	3 (27.3)	3 (42.9)	0	

IU = International unit.

* Data available for 35 of 45 patients.

† Data available for 30 of 45 patients.

‡ Data available for 11 of 45 patients.

The survivors were younger with a lesser frequency of symptoms (fever, cough, breathlessness, reduced appetite, anosmia, diarrhea, and body weakness) as shown in Tables 1 and 2. The non-survivor had more of abnormal chest findings and hypoxemia at presentation. Obesity and diabetes mellitus were more frequent among non-survivors. The non-survivors had long durations of symptoms before the presentation and a shorter hospital stay. Non-survivors had more of abnormal laboratory findings (Table 3).

The total in-hospital mortality was seven (15.6%), and all were males with severe and critical COVID-19. Five died of hypoxemic respiratory failure, and two died of MODS. Only two of the patients with respiratory failure were mechanically ventilated, and both died at 18 hours and 24 hours later. Of the two patients with renal impairment, one had hemodialysis and died later of MODS. After controlling for confounders, binary logistic regression showed the independent variables predicted of poor outcomes (death) were hypoxemia with an aOR of 2.541 (95% CI: 1.263–5.114) and serum creatinine level above 1.5 mg/dL, aOR of 4.301 (95% CI: 1.88–9.845) as shown in Table 4.

**Table 4:** Risk factors associated with mortality (in-hospital death)

Variable	Categories	*n*	Unadjusted OR (95% CI)	*P-*value	Adjusted OR (95% CI)	*P-*value
Age (years)	≥ 50	13	26.571 (2.744–257.280)	0.001	1.127 (0.605, 2.101)	0.706
	< 49^ref^	32				
Fever	Yes	17	0.025 (0.001–0.469)	0.014	–	–
	No^ref^	28				
Cough	Yes	13	0.038 (0.004–0.364)	0.005	–	–
	No^ref^	32				
Breathlessness	Yes	11	0.075 (0.012–0.481)	0.006	1.267 (0.667,2.406)	0.470
	No^ref^	34				
Reduced appetite	Yes	8	0.088 (0.014–0.550)	0.009	–	–
	No^ref^	37				
Diarrhea	Yes	6	0.114 (0.017–0.768)	0.026	–	–
	No^ref^	39				
Body weakness	Yes	6	0.114 (0.017–0.768)	0.026	–	–
	No^ref^	39				
Chest findings	Normal^ref^	34	115.000 (5.575–2,372.160)	0.002	1.034 (0.558–1.917)	0.916
	Abnormal	11				
Pulse rate	≤ 100	35	16.5 (2.492–109.270)	0.004	1.019 (0.599, 1.733)	0.946
	> 100^ref^	10				
Hypoxemia*	Yes	13	9.375 (1.525–57.621)	0.016	2.541 (1.263,5.114)	**0.009**
	No^ref^	32				
Diabetes mellitus	Yes	6	0.11 (0.017–0.768)	0.026	0.623 (0.298, 1.306)	0.210
	No^ref^	39				
White blood count (× 10^9^/L)	< 3.5	1	3.667 (0.116–115.805)	0.461	–	–
	3.5–10^ref^	29				
	> 10	5	9 (0.907–89.27)	0.094	–	
Neutrophils (%)	< 40	13	1 (0.019–54.160)	0.049	0.972 (0.404, 2.336)	0.949
	40–60^ref^	13				
	> 60	9	22.01 (1.020–483.284)		1.057 (0.509, 2.192)	0.883
Serum Cr (mg/dL)	≤ 1.5	33	108.333 (3.441–3,410.867)	0.005	4.301 (1.88–9.845)	**0.001**
	> 1.5^ref^	2				

OR = odds ratio, ref= reference value for the OR.

* At presentation.

## DISCUSSIONS

The mean age of this cohort is slightly higher than the mean age reported in an earlier Nigerian^15^ study, but lower than values reported from cohorts in other studies.^9,17^ This study also showed that about 50% of the cases were in the middle age-group. This finding is comparable with observations from cohorts in China.^12^ By contrast, most of the admitted cases in Europe cohorts were in older age-groups. The difference in age distribution between our study and the European^14^ study may be because of different cohorts. Our cohort involved all categories of cases compared with the European cohort that involved critically ill patients managed at the ICU. The import of the predominance of younger age found in this cohort compared with the other cohorts suggests the need to prioritize screening in the age-group, especially if the country is to curtail the spread of COVID-19. The male patients were more affected in this study, which is consistent with other studies and further affirm male gender is susceptible to SARS-CoV-2 infection.^18,19^ Male gender’s higher risk of COVID-19 has been attributed to the role of X-linked in the immune response and down-regulation of the angiotensin-converting enzyme 2 (ACE2) mRNA receptor, the receptor for SARS-CoV-2, by 17-beta estradiol in female gender.^20–23^ The most common symptoms in this cohort included fever, cough, and breathlessness, which are similar to reports from the other parts of the world.^8,9,24^ Most of our patients had normal liver function tests. By contrast, a meta-analysis showed that some COVID-19 cases had elevated aspartate aminotransferase, alanine aminotransferase, and reduced albumin.^25^ Although we could not make a definitive conclusion on the derangement of liver function tests because of the relatively small sample size, hepatic derangement may be uncommon. Elevated creatinine occurred in 5.7% of our cohort but less than 10% found among Chinese cohorts^18^ and suggested the need to look for renal impairment in adults with COVID-19 in our environment. The chest radiographic findings in this study are similar to the report of other researchers.^26^ The in-hospital mortality rate in this cohort is 15.6%, which falls within the reported case fatality rate range of 3.6–45% in the literature depending on the cohort of studies.^9,13,14,18,27^ Our findings show in-hospital mortality is about eight times higher than the country’s case fatality rate of 1.9%.^5^ One reason may be that those who have a severe form of the disease are the ones likely to present to a tertiary health care facility like ours because mild cases or asymptomatic cases may not present at the tertiary health facilities.^28^ This finding could also suggest that the death rate from COVID-19 may be far higher than being reported. Hence, there is a need to step up the various control measures put in place for early case identification and prompt intervention.

This study showed that the non-survivors had more symptoms with fever in all. By contrast, there were no differences in the symptoms between the survivors and non-survivors in Wuhan, China.^24^ We also observed that the pulse rate, abnormal chest findings, and hypoxemia were more among the non-survivors. These findings are evidence of systemic involvement, which predicts poor outcomes. The non-survivors in this cohort had a shorter hospital stay. Whereas studies from other cohort studies^29–31^ also showed shorter hospital stays among the non-survivors, the duration (days) before death was longer than that in this present cohort. The reasons for the shorter hospital stay in the present study than the cohorts outside Africa may be because of some reasons; we observed the late presentation in the non-survivors in our cohort and limited capacity to support advance care required in severe and critically COVID-19 cases. Leukocytosis with neutrophilia was noticed to be more common among non-survivors. These findings contrast with an earlier study from Wuhan in China, where there were more leukopenia and lymphopenia cases.^18^ To support our observation is the work of Wang et al.^26^ who also reported leukocytosis, neutrophilia, and lymphopenia. The observation of leukocytosis among the non-survivor in this cohort may reflect the high prevalence of infections in Africa, with the likelihood of early bacteremia complicating the viral disease.

After adjusting for confounders, the independent predictors of death were hypoxemia and elevated creatinine above 1.5 mg/dL, which were similar to other reports. In Wuhan, China, Xie et al.^32^ also identified hypoxemia as an independent predictor of mortality in COVID-19 patients. The pathophysiology of hypoxemia-related death in COVID-19 patients involved exacerbation of inflammatory cytokines and immune response with a worsening cytokine storm which further led to lung injury and persistent hypoxemia and ultimate poor outcomes.^33^ In low- and middle-income countries, a handheld portable pulse oximeter, which is cheap and affordable, may help in early screening to identify patients with hypoxemia, prompting early intervention. Elevated creatinine, which shows renal impairment, was identified in a recent review to be associated with an increase in mortality in COVID-19 patients.^34^ The pathophysiology of poor outcomes in the presence of renal impairment may be related to down-regulation of ACE2 in response to increasing angiotensin II because of acute lung injury with subsequent worsening of acute kidney injury.^35^

### Limitations.

Although we could retrieve the case records of all the patients managed during the period, a few had incomplete investigations. Thus, only 35 had full blood counts, 30 had liver function tests, and 11 had chest radiographs among the patients. Besides, these are hospital-based data that represented most cases from moderate to severe COVID-19 who presented to our facility and may not be an accurate reflection of the actual burden of COVID-19 in the state.

## CONCLUSION

Our study showed that hypoxemia and elevated creatinine were independent predictors of mortality in patients with COVID. In-hospital mortality in our study was eight times the country’s case fatality rate. Leukocytosis with lymphopenia and neutrophilia is the main laboratory finding, although they did not predict mortality.
